# Electroconvulsive Therapy for Malignant Catatonia: A Case Report in the Intensive Care Unit

**DOI:** 10.7759/cureus.83714

**Published:** 2025-05-08

**Authors:** Maria Beatriz Dias Vieira, Joel Pinto, Cristiana Madaíl Grego, Mónica Almeida, Eduardo Santos Ribeiro

**Affiliations:** 1 Intensive Care Unit, Unidade Local de Saúde da Região de Aveiro, Aveiro, PRT; 2 Psychiatry, Unidade Local de Saúde da Região de Aveiro, Aveiro, PRT

**Keywords:** cardiorespiratory arrest, catatonia, critical illness, electroconvulsive therapy, intensive care unit, malignant catatonia, mania

## Abstract

Catatonia is a complex neuropsychiatric syndrome characterized by motor and behavioral disturbances, which can complicate both psychiatric and medical conditions. Diagnosing catatonia in the Intensive Care Unit (ICU) can be challenging due to symptom overlap with other conditions, such as delirium. Electroconvulsive therapy (ECT) may be considered in certain cases, particularly when standard treatments fail, though its use in ICU settings is rare due to concerns about patient stability and procedural risk. We present the case of a 43-year-old male, admitted for a manic episode, who was transferred to the ICU following a cardiorespiratory arrest and subsequently treated with ECT for malignant catatonia.

## Introduction

Catatonia is a form of acute brain dysfunction characterized by a marked inability to initiate or control voluntary movements, despite having the physical capacity to do so [1]. When associated with severe autonomic abnormalities, it may require admission to the Intensive Care Unit (ICU) [2]. This syndrome has diverse clinical presentations and, like delirium, may include hypoactivity, hyperactivity, or abnormal behavior [3].

In order to diagnose catatonia, three or more of the symptoms listed in the Diagnostic and Statistical Manual of Mental Disorders (Fifth Edition) are required, including stupor, waxy flexibility, catalepsy, mutism, posturing, negativism, stereotypes, mannerisms, grimacing, agitation, echopraxia, and echolalia [4]. In clinical practice, the Bush-Francis Catatonia Rating Scale is commonly used to assess the severity of these symptoms and monitor treatment response. This scale, which evaluates 23 items related to motor behaviors, psychological symptoms, autonomic signs, and repetitive movement patterns, helps classify catatonia as mild, moderate, or severe based on the total score [5].

The subtypes of catatonia exist on a continuum between retarded and excited, and any of these behavioral phenotypes can progress to what’s called malignant catatonia (MC), a life-threatening and rapidly progressive condition characterized by fever, autonomic instability (including hyper/hypothermia, brady/tachycardia, hypo/hypertension, and hypo/hyperpnea), delirium, and rigidity [6]. Some common, yet nonspecific, laboratory findings in MC include leukocytosis, elevated creatine kinase, and low serum iron [7]. If promptly diagnosed, MC can be managed effectively in up to 80% of patients with benzodiazepines and electroconvulsive therapy (ECT) [3]. However, in the ICU setting, despite its potential benefits, the use of ECT remains relatively rare, with only a few cases reported [8,9].

## Case presentation

A 43-year-old male with no prior medical history presented to the Emergency Department with five days of bizarre behavior, confused speech, and psychomotor agitation. His housemate reported episodes of agitation, heteroaggressiveness, and disrupted sleep, with the patient claiming to be Adam and Eve, performing counting rituals, and exhibiting animal-like movements and sounds. He was given haloperidol and chlorpromazine, which calmed him. A non-contrast cranial computed tomography (CT) scan and blood tests, including drug and ethanol screening, showed no abnormalities (Table 1).

**Table 1:** Admission blood tests within normal reference ranges ALT: alanine aminotransferase; AMP: amphetamine; AST: aspartate aminotransferase; aPTT: activated partial thromboplastin time; Ca²⁺: calcium; CK-MB: creatine kinase-MB; Cl⁻: chloride; COC: cocaine; CRP: C-reactive protein; ESR: erythrocyte sedimentation rate; HCO₃⁻: bicarbonate; INR: international normalized ratio; K⁺: potassium; Na⁺: sodium; PT: prothrombin time; T4: thyroxine; TSH: thyroid-stimulating hormone; WBC: white blood cell count

Parameter	Result	Reference range
Hemoglobin	14.6 g/dL	13.0 - 18.0 g/dL
Hematocrit	44.4%	41.0 - 55.0%
WBC	10.1 x 10^9^/L	4.1 - 11.1 x 10^9^/L
Platelet count	270 x 10^9^/L	150 - 500 x 10^9^/L
Serum urea	22.1 mg/dL	19.0 - 51.0 mg/dL
Creatinine	0.74 mg/dL	0.70 - 1.30 mg/dL
Na⁺	137.45 mmol/L	132.00 - 146.00 mmol/L
K⁺	3.90 mmol/L	3.50 - 5.50 mmol/L
Cl⁻	104.61 mmol/L	99.00 - 109.00 mmol/L
HCO₃⁻	25.4 mmol/L	22.0 - 28.0 mmol/L
Ca^2+^	1.18 mmol/L	1.13 - 1.32 mmol/L
AST	33 U/L	13 - 38 U/L
ALT	32 U/L	10 - 49 U/L
Alkaline phosphatase	90 U/L	46 - 116 U/L
Total bilirubin	0.84 mg/dL	0.30 - 1.20 mg/dL
Direct bilirubin	0.23 mg/dL	<0.30 mg/dL
Blood glucose level	130 mg/dL	65 - 110 mg/dL
Troponin I	11.1 pg/mL	<47.3 pg/mL
CK-MB	200 U/L	46 - 171 U/L
TSH	0.94 mU/L	0.55 - 4.78 mU/L
Free T4	1.26 ng/dL	0.80 - 1.80 ng/dL
INR	0.99	0.70 - 1.30
PT	13.2 seconds	11.7 - 15.3 seconds
aPTT	30.0 seconds	24.8 - 34.4 seconds
CRP	0.09 mg/dL	0.00 - 0.50 mg/dL
ESR	11 mm	<15 mm
pH	7.384	7.350 - 7.450
pCO₂	43.5 mmHg	35.0 - 45.0 mmHg
pO₂	94.6 mmHg	80.0 - 100.0 mmHg
Lactate	0.8 mmol/L	<1.8 mmol/L
Ethanol	<10.0 mg/dL	<10.0 mg/dL
Amphetamine	Negative	Negative
Tricyclic antidepressants	Negative	Negative
Benzodiazepines	Negative	Negative
Cocaine	Negative	Negative
Methadone	Negative	Negative
Methamphetamine	Negative	Negative
3,4-methylenedioxymethamphetamine	Negative	Negative
Tetrahydrocannabinol	Negative	Negative
Morphine	Negative	Negative
Barbiturates	Negative	Negative

Suspecting a manic episode, the patient was admitted to the Psychiatry Department for stabilization and started on lorazepam and olanzapine.

**Figure 1:**
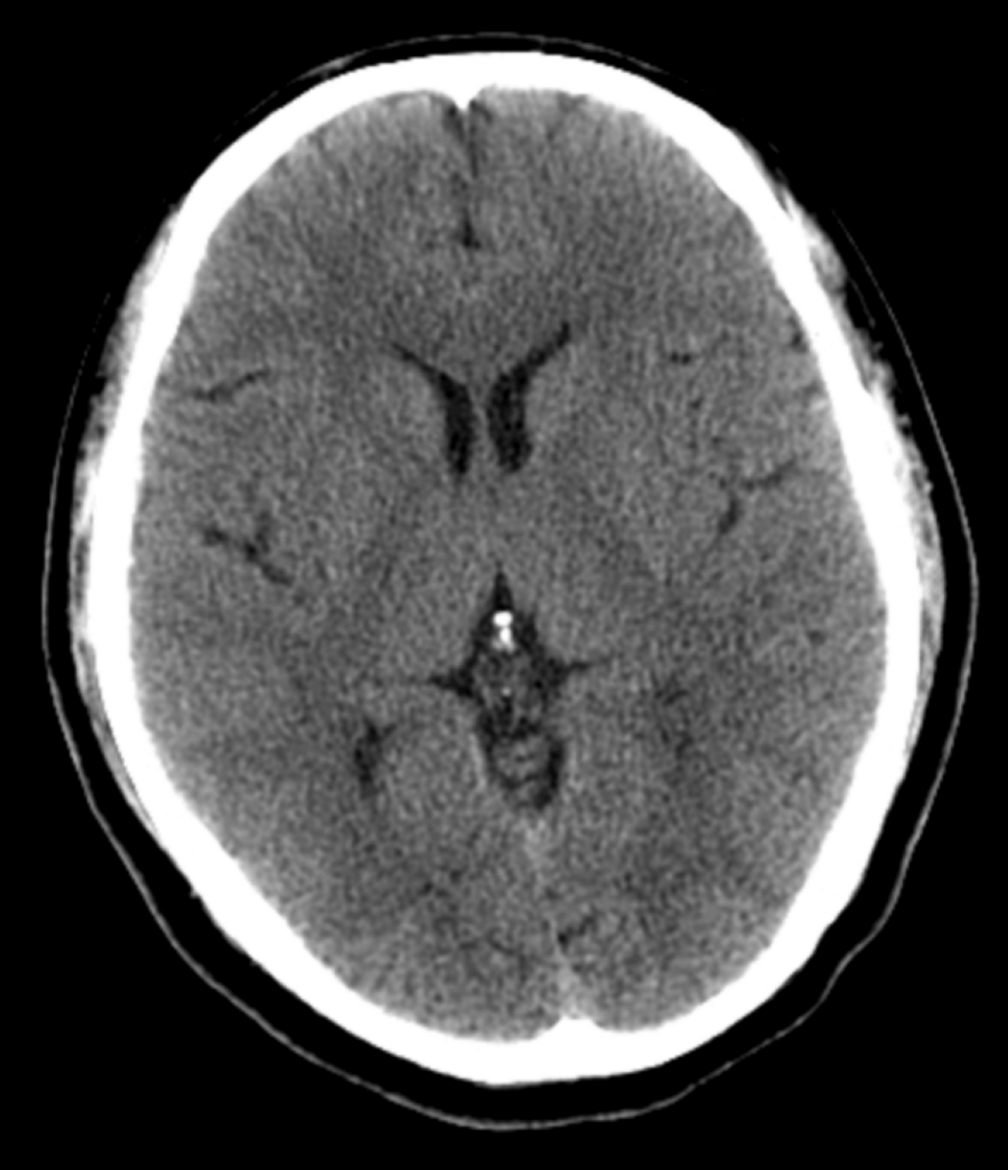
Non-contrast cranial CT scan revealed no acute lesions following the cardiorespiratory arrest CT: computed tomography

On the second day, the patient was found in cardiorespiratory arrest in asystole, which was reversed after one cycle of advanced life support with epinephrine. Following resuscitation, his Glasgow Coma Scale score was 3, with fixed, dilated pupils, hypertension, and tachycardia. Flumazenil was administered in an attempt to reverse a possible benzodiazepine effect, without improvement in consciousness. He was intubated and placed on mechanical ventilation. Arterial blood gas analysis revealed severe hypokalemia (2.8 mEq/L), which was corrected. A repeat non-contrast cranial CT scan revealed no acute lesions (Figure 1).

The patient was admitted to the ICU for post-cardiorespiratory arrest care and neurological monitoring. He was kept sedated with propofol and remifentanil and underwent a lumbar puncture, which showed normal cerebrospinal fluid (CSF) (Table 2).

**Table 2:** Cerebrospinal fluid analysis showing normal results AFB: acid-fast bacilli; BK: bacillus of Koch; HSV: herpes simplex virus; PCR: polymerase chain reaction

Parameter	Result	Reference range
Glucose	81.5 mg/dL	40.0 - 80.0 mg/dL
Total protein	28.5 mg/dL	15.0 - 45.0 mg/dL
Albumin	11.4 mg/dL	12.0 - 26.0 mg/dL
Cell count	1.6/mm³	-
Direct exam (Gram stain)	No microorganisms observed	-
Culture	Negative	-
BK direct exam (AFB stain)	No acid-fast bacilli observed	-
*HSV* by molecular testing	Negative	-
*Cryptococcus gattii* (PCR)	Negative	-
HSV-1	Negative	-
HSV-2	Negative	-
Enterovirus	Negative	-
Human parechovirus	Negative	-
Human herpes virus 6	Negative	-
Varicella zoster virus	Negative	-
Escherichia coli K1	Negative	-
Haemophilus influenzae	Negative	-
Listeria monocytogenes	Negative	-
Neisseria meningitidis	Negative	-
Streptococcus pneumoniae	Negative	-
Streptococcus agalactiae	Negative	-
Mycoplasma pneumoniae	Negative	-
Streptococcus pyogenes	Negative	-
Cryptococcus gattii	Negative	-

At 72 hours of neuroprotection, sedative and analgesic medications were discontinued, with the patient exhibiting marked agitation, persistent coughing, and autonomic instability (hypertension and tachycardia), without eye opening or following commands. In this context, pharmacological adjustments were made, maintaining propofol, suspending remifentanil, initiating fentanyl and dexmedetomidine, and reintroducing lorazepam and olanzapine. Concurrently, the patient developed a fever (maximum temperature of 38.2°C), and samples of urine, tracheobronchial secretions, and blood were collected for microbiological analysis.

In the following days, daily attempts were made to reduce and discontinue the infusions of propofol and fentanyl, with the patient failing to awaken and presenting marked hypertension, tachycardia, and sweating. After the isolation of *Klebsiella pneumoniae* in the culture of tracheobronchial secretions and the absence of radiographic findings consistent with pneumonia, a diagnosis of tracheobronchitis associated with orotracheal intubation was made, and ertapenem was initiated, which was later de-escalated to cefepime based on antimicrobial susceptibility testing.

In the subsequent days, there was continued difficulty in weaning the sedation, with the patient tolerating very short periods of mechanical ventilation with pressure support due to marked motor agitation, hyperpnea, hypertension, and frequent coughing. Despite targeted antibiotic therapy and a reduction in inflammatory biomarkers, the patient continued to experience fever spikes.

**Figure 2:**
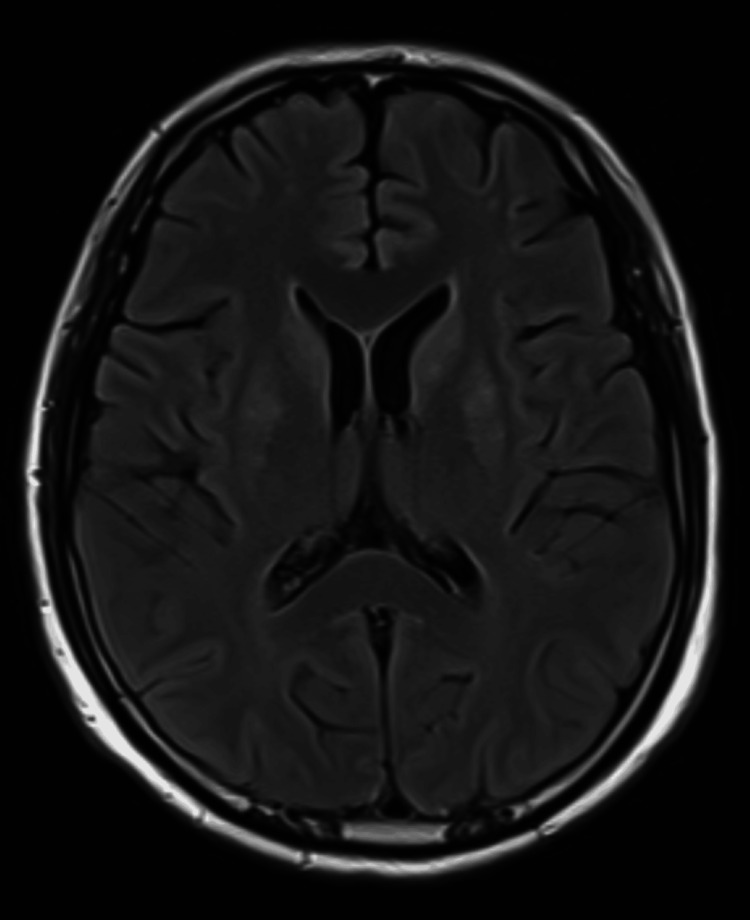
T2-FLAIR-weighted cranial MRI excluded hypoxic-ischemic lesions secondary to cardiorespiratory arrest FLAIR: fluid-attenuated inversion recovery; MRI: magnetic resonance imaging

The patient underwent a cranial magnetic resonance imaging (MRI), which ruled out the presence of possible hypoxic-ischemic lesions resulting from the cardiorespiratory arrest and that could explain the neurological condition (Figure 2).

**Figure 3:**
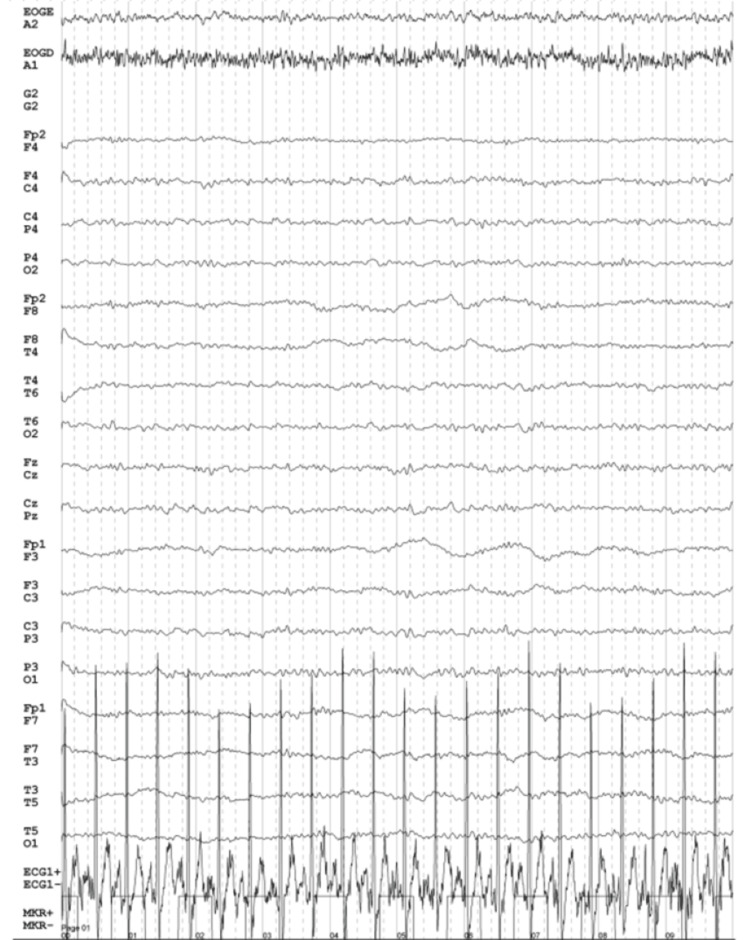
Patient's EEG findings showing a preserved baseline rhythm and no epileptiform activity EEG: electroencephalogram

An electroencephalogram (EEG) was also performed which ruled out epileptiform activity (Figure 3).

Psychiatry was consulted, and the antipsychotic therapy was adjusted, with the discontinuation of lorazepam and the initiation of haloperidol and oxazepam. Given the difficulty in achieving a neurologically stable state for safe extubation with prolonged ventilatory weaning, the patient underwent a surgical tracheostomy.

After pharmacological adjustment and daily reduction of sedation, the patient was more alert but not making eye contact, with upper limb spasticity and a tendency to maintain a fetal position. He also presented cervical rotation movements, hyperextension with rigidity, and repeated lower limb movements with rigidity. Additionally, the patient continued to experience fever spikes, tachycardia, and hypertension despite antihypertensive therapy, along with excessive sweating. 

Neurology was involved in the case, and autoimmune encephalitis was considered among the differential diagnoses. A repeat lumbar puncture and serological testing were conducted for antibodies and target antigens analysis, both of which returned negative results (Table 3).

**Table 3:** Negative results from cerebrospinal fluid analysis and serological testing for autoimmune encephalitis AMPA: α-amino-3-hydroxy-5-methyl-4-isoxazolepropionic acid; CASPR2: contactin-associated protein-like 2; CRMP5: collapsin response mediator protein 5; CSF: cerebrospinal fluid; DNER: delta and notch-like epidermal growth factor-related receptor; EGF: epidermal growth factor; GABA: gamma-aminobutyric acid; GAD67: glutamic acid decarboxylase isoform 67; GFAP: glial fibrillary acidic protein; IgG: immunoglobulin G; IgLON5: immunoglobulin-like cell adhesion molecule 5; LGI1: leucine-rich glioma-inactivated 1; NMDA: n-methyl-d-aspartate; PNMA2: paraneoplastic antigen Ma2; Ri: Nova1-related antigen; SOX1: sry-box transcription factor 1; Tr: testis-restricted; VGKC: voltage-gated potassium channel; Yo: cdr2-related antigen; Zic4: zinc finger protein of the cerebellum 4

Antibody tested	Target antigen	Serum result	CSF result
Kelch-like protein 11 antibodies	Kelch-like protein 11	Negative	Negative
Neuronal antibodies	Hu, Yo, Ri, CV2 (CRMP5), PNMA2, amphiphysin, recoverin, SOX1, titin	Negative	Negative
Tr (DNER) antibodies	Delta and notch-like EGF-related receptor	Negative	Negative
GFAP antibodies	Glial fibrillary acidic protein	Negative	Negative
Neurexin-3 alpha antibodies	Neurexin-3 alpha	Negative	Negative
Zic4 antibodies	Zinc finger protein of the cerebellum 4	Negative	Negative
IgLON5 antibodies	Immunoglobulin-like cell adhesion molecule 5	Negative	Negative
VGKC complex antibodies	Voltage-gated potassium channel complex	17 pmol/L	<5 pmol/L
GAD67 antibodies	Glutamic acid decarboxylase 67	Negative	Negative
CRMP5 (CV2) antibodies	Collapsin response mediator protein 5	Negative	Negative
NMDA receptor antibodies (IgG)	N-methyl-D-aspartate receptor	Negative	Negative
AMPA1 receptor antibodies (IgG)	AMPA receptor subtype 1	Negative	Negative
AMPA2 receptor antibodies (IgG)	AMPA receptor subtype 2	Negative	Negative
GABA-A receptor antibodies (IgG)	Gamma-aminobutyric acid A receptor	Negative	Negative
GABA-B receptor antibodies (IgG)	Gamma-aminobutyric acid B receptor	Negative	Negative
CASPR2 antibodies (IgG)	Contactin-associated protein-like 2	Negative	Negative
LGI1 antibodies (IgG)	Leucine-rich glioma-inactivated 1	Negative	Negative
Glycine receptor antibodies	Glycine receptor	Negative	Negative

In the absence of clinical improvement, Psychiatry was reconsulted, and MC was considered as a potential diagnosis. Given the previous lack of favorable response to lorazepam, the patient was subjected to a series of ECT sessions, with a two-day interval between them. After the second session, the patient showed significant improvement in the neurological state, remaining awake without sedation, making eye contact, and smiling when approached. After the third session, the patient began and tolerated periods of sitting in a chair, still experiencing transient, self-limited episodes of hypertension and tachycardia. After the fourth session, the patient tolerated periods of spontaneous ventilation, started following simple commands, and showed improved muscle tone with tolerance of functional rehabilitation and physical therapy. The patient underwent a fifth ECT session and was decannulated one day later. 

Approximately two months after ICU admission, the patient was discharged to the Psychiatry ward, where the ECT sessions were maintained. He was alert, attentive, cooperative, and communicative, with fluent and coherent speech, following commands, pain-free, with less pronounced spasticity, dysphagia for liquids, and able to walk short distances with assistance.

## Discussion

Despite being initially described in association with severe psychiatric disorders, such as schizophrenia and bipolar disorder, catatonia is now recognized as a consequence of many other underlying conditions, including infections, metabolic disturbances, mood, rheumatologic, and neurological disorders, as well as substance use or withdrawal [4]. Particularly in the ICU setting, malnutrition, dehydration, the administration or abrupt discontinuation of dopamine antagonists, such as haloperidol and metoclopramide, agents with serotonergic activity, like tramadol and linezolid, and the use of antipsychotics for delirium management without concomitant benzodiazepines may trigger or aggravate MC [3].

However, as was done in this case, it is essential to differentiate MC from other critical conditions that may present with overlapping symptoms, such as rigidity, autonomic instability, altered mental status, and fever, but differ significantly in pathophysiology and treatment. Key differential diagnoses include neuroleptic malignant syndrome (NMS), serotonin syndrome, nonconvulsive status epilepticus (NCSE), and autoimmune encephalitis [3]. Differentiating NMS from MC is particularly challenging, as both share several clinical features; however, NMS is more directly associated with the use of antipsychotic agents [10]. In contrast, serotonin syndrome typically resolves within three to five days after discontinuation of serotonergic medications and often includes hyperreflexia and clonus [11]. NCSE requires a high index of suspicion and confirmation via EEG, as its clinical presentation may closely mimic catatonia [12]. Lastly, autoimmune encephalitis, particularly anti-NMDA (N-methyl-D-aspartate) receptor encephalitis, may manifest with catatonia-like features. Early recognition using CSF analysis, brain MRI, EEG, and antibody testing is crucial, as management relies on immunotherapy rather than benzodiazepines or ECT [13]. Moreover, some authors propose that MC may, in certain cases, represent a drug-induced syndrome precipitated by conditions such as NMS or serotonin syndrome [3]. 

In this context, the pathophysiology behind catatonia is still unclear, although some studies suggest it may result from dysregulation in the brain’s motor circuits, especially in the cortico-thalamic motor pathway, neurotransmitter alterations, with reduced gamma-aminobutyric acid (GABA) activity in the sensorimotor cortex, glutamate hyperactivity in the basal ganglia, and a dopamine D2 receptor blockade, as well as pro-inflammatory states with cytokine release and acute phase response [3,9,14]. Evidence suggests a potential genetic predisposition to catatonia, particularly linked to genes on chromosomes 15 and 22 [15]. 

The reported prevalence of catatonia in ICU patients is still unclear, but studies suggest that up to a third of patients with delirium due to medical or surgical-related critical illness will develop catatonia during their stay [3]. This prevalence is particularly high in patients with severe psychiatric comorbidities, neurological disorders, or those who are mechanically ventilated. A study of 136 patients treated in an ICU with mechanical ventilation and/or vasopressors found that catatonia was present in approximately 35% [16]. However, this syndrome is severely underdiagnosed in this setting, mainly due to uncertainty regarding assessment and diagnostic criteria, especially when it comes to the extent to which autonomic abnormalities may actually be attributable to catatonia [17]. 

Regarding treatment, benzodiazepines are the first-line therapy for catatonia, with lorazepam being the most commonly used. Remission rates of up to 80% have been reported, particularly when administered intravenously, given the frequent impairment of enteral absorption in critically ill patients [3,6,9]. Other benzodiazepines, like diazepam, oxazepam, and clonazepam, as well as the benzodiazepine agonist zolpidem, have also proven effective in the treatment of catatonia [3]. However, these drugs should be used cautiously in patients with overlapping delirium, since they may exacerbate their symptoms and enhance their sedative properties [18]. 

ECT is a well-established treatment for numerous psychiatric diseases, such as schizophrenia, schizoaffective disorder, bipolar disorder, and severe depression [4]. Currently, it is considered the definitive treatment for catatonia when benzodiazepines fail, with some authors suggesting the concomitant use of both therapies as more beneficial [3]. Even though the mechanism behind the use of ECT in catatonia is not completely understood, it seems to work by increasing the deficient GABAergic signaling and receptor expression [19]. The response rates of ECT in these cases can reach 80% to 100%, as long as it is initiated within a five-day window from the diagnosis of MC [4]. It’s currently unknown what the number of ECT sessions needed to reach remission is, with some authors suggesting at least six to eight consecutive sessions as an optimal number for clinical improvement [3,4].

While still relatively uncommon in ICU settings, the use of ECT in critically ill patients requires careful case selection to ensure both safety and efficacy. Key considerations include the underlying etiology of catatonia, the severity of the patient’s medical condition, and the presence of any contraindications to ECT [4]. Potential risks, such as arrhythmias, cardiovascular instability, and increased intracranial pressure, must be carefully weighed against the expected benefits [16]. When properly managed, ECT has demonstrated substantial clinical benefit in this patient population [3]. Furthermore, in the ICU setting, ECT has also been shown to be safe and effective in some cases of refractory delirium, particularly when catatonic features are present [16].

In this particular clinical case, it is evident that the recent diagnosis of a psychiatric disorder, coupled with the underlying ICU-related conditions (including infection, mechanical ventilation, and medication), contributed to a clinical presentation consistent with MC. Several factors associated with a favorable response to ECT, as identified by some authors, were present in this case, including the patient’s young age, the presence of autonomic dysfunction, and the potential existence of an underlying mood disorder [3].

## Conclusions

MC is a life-threatening neuropsychiatric condition that may evolve rapidly and present with severe systemic manifestations, such as autonomic instability. With early recognition and appropriate treatment, including benzodiazepines and ECT, MC can be effectively managed. This report emphasizes the importance of maintaining a high index of suspicion for catatonia in critically ill patients and illustrates that ECT may be considered as a therapeutic option, even in complex ICU settings. This decision requires careful consideration of risks and benefits, and close collaboration among multidisciplinary teams is essential for navigating the diagnosis and treatment options in these challenging cases.
